# Sun Exposure during Water Sports: Do Elite Athletes Adequately Protect Their Skin against Skin Cancer?

**DOI:** 10.3390/ijerph18020800

**Published:** 2021-01-19

**Authors:** Guillermo De Castro-Maqueda, Jose V. Gutierrez-Manzanedo, Carolina Lagares-Franco, Magdalena de Troya-Martin

**Affiliations:** 1 Department of Physical Education, Faculty of Education Sciences, University of Cádiz, 11510 Cádiz, Spain; guillermoramon.decastro@uca.es; 2 Department of Statistics and Operational Research, University of Cádiz, 11510 Cádiz, Spain; carolinalagares@uca.es; 3 Dermatology Department, Costa del Sol Hospital, 29603 Marbella, Spain; magdalenatroya@gmail.com

**Keywords:** sun protection, skin cancer, sunscreen use, sun exposure, water sports

## Abstract

Excessive sun exposure is the main avoidable cause of skin cancer. Outdoor sports performed without adequate photoprotection are risky practices in this respect. This study examines whether elite athletes in water sports (specifically surfing, windsurfing, and sailing) take appropriate measures to protect their skin from the sun, and whether there are differences in this respect according to age, gender, or sports discipline. This study is based on a questionnaire-based health survey. World championship competitors completed a self-administered questionnaire on their sun protection and exposure habits, as well as sunburns during the last sports season. In total, 246 participants, with an age range of 16–30 years, completed the questionnaire. Of these, 49.6% used inadequate sun protection. Those who protected their skin appropriately tended to be older than those who did not (average age = 23.28 and 20.69 years, respectively; *p* = 0.000). There were no significant differences in sun protection habits between male and female athletes. The rate of sunburn was very high (76.7%). A high proportion (22.5%) of participants never used sunscreen. Elite athletes in water sports are at real risk of skin lesions from overexposure to the sun, associated with inadequate photoprotection practices. Campaigns to raise awareness and to promote the early detection of skin cancer should target these risk groups.

## 1. Introduction

Skin cancer is one of the most-rapidly proliferating diseases in Europe and worldwide [1,2]. In Spain and the United States, for example, the incidence has tripled in recent decades, despite the prevention campaigns carried out [3,4]. Exposure to ultraviolet radiation is known to be one of the greatest environmental risk factors for skin cancer. Nevertheless, this is considered one of the most preventable types of cancer.

Australia, which has the world’s highest mortality rate from skin cancer, was one of the first countries to seek to raise awareness of the need to protect the skin from damage caused by prolonged solar exposure and inadequate protection against UV radiation [5].

The rising incidence of skin cancer has been attributed to changes in lifestyles, with many populations receiving greatly increased intermittent exposure to the sun due to the increasing popularity of open-air leisure and recreation activities [6]. Moreover, in many cases these activities are being performed at an ever-earlier age. Another significant factor is the popularity of sunbathing, since the fashion of tanning began in the 1960s. Nevertheless, this trend seems to have decreased in recent years, to the extent that solar risk awareness campaigns have penetrated the population [7]. However, unless appropriate protective measures are taken against UV radiation, sunbathing can be a major cause of skin cancer.

The lack of awareness among young people about the dangers of inadequate sun protection habits is an additional risk factor for skin cancer.

The use of sunscreen is an effective means of reducing the incidence of squamous cell carcinoma, melanocytic nevi, and solar keratoses, among other skin lesions [8,9]. This is particularly important for athletes, as the performance of outdoor physical exercise and sports activities has been related to the development of skin cancer, including malignant melanoma and basal cell carcinoma [6,10,11].

To our knowledge, few studies have been made of photoprotection behaviour during water sports, and fewer still with respect to elite athletes, although the latter may be role models for young people, who are likely to be less aware of the dangers and make less use of photoprotection measures than adults [12]. The aim of our study is to examine the sun exposure and protection habits of elite athletes in water sports, according to their age, gender, and sports discipline.

## 2. Materials and Methods

This cross-sectional questionnaire-based study of the sun exposure and protection practices of elite athletes in water sports, based on a convenience sample. All data were recorded anonymously and treated in strict compliance with Spanish data protection laws (Law 41/2002 of 14 November and Law 15/1999 of 13 December).

### 2.1. Study Population

A total of 246 professional athletes, aged 16–30 years, participating in world championship competitions in the disciplines of surfing, windsurfing, and sailing, were invited to take part in this study, which took place between September 2012 and July 2014.

The athletes in the study sample were chosen for convenience and asked to fill out a health questionnaire on their photoprotection and sun exposure habits during sports practice [13,14]. Those under 18 years of age were required to provide parental consent to participate in the study. The questionnaires were distributed and filled out before the competition event or training session.

The questionnaires were completed in the presence of a research interviewer, who was available to resolve possible doubts about interpretation of the questionnaire items. Subsequently, a dermatologist performed a complete skin check of each participant, using a dermatoscope to detect potentially malignant lesions. When necessary, the athletes were referred to their specialists for treatment and follow-up.

Each participant was given an information booklet and a sample of sunscreen, and was informed about the real risk they faced as elite athletes in water sports who are exposed to the sun for many hours during their training and competitions.

The inclusion criteria applied in this study were that the athletes should be aged 16–30 years, be participating in the world championship of their sports discipline, and have signed the consent form to take part in the study. Finally, in every case the questionnaire should be filled out completely and correctly.

### 2.2. Questionnaire

The two-page questionnaire used for this study was health-focused and prepared by experts in the field of photoprotection and sun exposure [14]. The same questionnaire has been used in previous research [15,16,17]. The following information was obtained for each participant: age (years), sex, duration of sun exposure per day (hours and minutes), type of sport performed (surfing, wind surfing, or sailing), and nationality. Information on participants’ sun exposure and protection habits (the use of sunscreen, wearing a cap or hat, long-sleeved t-shirt and/or sunglasses, and staying in the shade whenever possible) [14] was also requested (Supplementary Material File S1).

The questionnaires were designed to be completed in 5–10 min, and the researcher was present throughout to clarify doubts and to ensure that the procedure was carried out consistently.

The questionnaire was printed in two languages, English and Spanish. The main variables for photoprotection and sun exposure practices were addressed in the following sections:

#### 2.2.1. Section One

1. Sun exposure: average time (hours) spent in the sun between 10 a.m. and 4 p.m., on weekdays and over the weekend;
2. Photoprotection (six practices recommended by the World Health Organization): use of sunscreen, wearing a long-sleeved t-shirt, wearing a cap or hat, staying in in the shade, wearing sunglasses, and avoiding sunbathing merely to get a tan. The responses were scored on a five-point Likert scale (1 = never; 2 = hardly ever; 3 = sometimes; 4 = often; 5 = always);
3. A history of sunburn episodes during the last sports season, scored on a six-item scale from 0 to 5 or more. Sunburn was defined as the presence of blistering and/or reddening and/or pain, lasting more than one day;
4. Skin colour: colour of skin not exposed to sunlight, on a six-category scale based on the Fitzpatrick skin phototypes [18]: pale (Type I); fair (Type II); darker white (Type III); light brown (Type IV); brown (Type V); dark brown or dark (Type VI).

#### 2.2.2. Section Two

1. Skin check-up: the athletes were asked if they had ever had a medical check-up of their skin; if so, they were asked to indicate when this had last been performed (month and year). They were also asked if they had examined their skin during the past year, including their back, to search for spots or lesions (or had received help in this task). If they had, they were asked to state the number of times this had been done.

#### 2.2.3. Section Three

1. Sports training routine: for this aspect, the athletes were asked how many hours they trained outdoors every day, on average; whether they used sunscreen on their face during training (and if so, the sun protection factor of the lotion); and whether they reapplied this sunscreen throughout the day [14,19].

### 2.3. Ethical Considerations

The study protocols for this project were reviewed and approved by the Institutional review board where the investigators were employed at the time of the study, namely the Medical Ethics Committee of the Dermatology Service, Costa del Sol Hospital (Marbella, Spain). All participants provided signed, informed consent before the start of the study.

### 2.4. Statistical Analysis

Descriptive analyses were performed, with measures of central tendency and dispersion for the quantitative variables, and of frequency distribution for the qualitative ones. Chi-square analyses were conducted to assess differences in photoprotection habits according to sports discipline and gender, as well as by effectiveness (high, medium, or low). Kolmogorov–Smirnov analyses were conducted to verify the normal distribution or otherwise of the quantitative variables. When the test results were not statistically significant, a Student’s *t*-test or ANOVA test was applied. If the variable did not follow a normal distribution, the Wilcoxon or the Kruskal–Wallis test was performed. All statistical tests were performed using IBM SPSS 2.0 (IBM, Armonk, NY, USA), and significance was assumed at *p* ≤ 0.05.

### 2.5. Measures

Three outcome variables were analysed—photoprotection practices, solar exposure, and sunburn events—by the participants during their last sport season.

Photoprotection practices—use of sunscreen, wearing a long-sleeved t-shirt, wearing a cap or hat, staying in the shade, and wearing sunglasses—were used to classify each participant’s photoprotection habits as either adequate or inadequate, assigning a composite score for these five forms of protection of ≥2.5 or <2.5 points. This composite score was computed by adding the answers to all five items on the 1–4 point ordinal scale (never/rarely = 1 point; sometimes = 2 points; often = 3 points; always = 4 points) and dividing by 5, according to previous studies [10,20].

## 3. Results

The questionnaire was completed by a total of 246 elite athletes in water sports from 30 different countries (135 from Europe and 105 from the rest of the world) who took part in world championship competitions in their respective disciplines.

Of these, however, six did not answer the questionnaire in full and were excluded. The final sample, therefore, was composed of 240 competitors, of whom 171 were men (71.3%) and 69 were women (28.8%), with an average age of 22 years (SD 5.8).

Of these participants, 32.9% were surfers (55 men and 24 women, average age = 23.3 years). Another 20% were windsurfers (36 men and 12 women, average age = 25.6 years), and 47.1% were sailors (80 men and 33 women, average age = 19.3 years). All were competing in their respective world championships, and on average spent four hours a day in the sun while training.

In terms of the Fitzpatrick classification [18], the athletes presented the following distribution of skin phototypes: Type I = 6.3%; Type II = 3.3%; Type III = 22%; Type IV = 32.5%; Type V = 9.2%; Type VI = 1.7%.

### 3.1. Photoprotection

A high proportion (49.6%; *n* = 119, 79 men and 40 women) of the athletes reported inadequate photoprotection habits. There were no significant differences in this respect either by gender or by nationality (Table 1).

### 3.2. Solar Exposure during the Last Sports Season

The duration of solar exposure during daily training/competition varied according to the sport practiced (Table 2). The sailors reported the longest average exposure (4 h 51 min), followed by the surfers (4 h 35 min) and the windsurfers (3 h 53 min).

### 3.3. Sunburn Events during the Last Sporting Season

With respect to the number of sunburn events during the last season, there were significant differences according to the sport in question (*p* < 0.002), for men and women alike, among the three sports analysed, with the surfers being most commonly affected (86.1% of the men and 83.3% of the women). Only 23.3% of the participants had never been burned (Table 3).

A significant number of participants had never used sunscreen (22.5%, *n* = 54). In this respect, there were no significant differences between women and men, or for any of the sports disciplines. The surfers reported the highest rates of use (91.1%, *n* = 72). There were no significant differences between men and women in regards to the reapplication of sunscreen.

### 3.4. Photoprotection Habits According to the Age of the Participants

The participants whose photoprotection habits were considered adequate had an average age of 23.28 years (SD = 6.03), while those whose protection was inadequate had an average age of 20.69 years (SD = 5.4). This age difference was statistically significant (*p* = 0.000), and so younger athletes are less likely to protect their skin sufficiently from the dangers of solar exposure (Table 4).

## 4. Discussion

To our knowledge, this is the first study conducted to determine the sun exposure habits and the adequacy of photoprotection practices of elite athletes in water sports. Our results highlight the considerable risk of skin cancer to this population, which suffers high rates of sunburn, with 76.7% of participants reporting at least one sunburn during the last sports season, while 27.5% reported three or more such events. This was especially true of the windsurfers, of whom 86.1% of men and 83.3% of women had had at least one sunburn during the last summer. Relative youth is an aggravating factor, with most sunburn episodes being suffered by those aged 20–25 years.

Overall, a high proportion of these athletes used inadequate measures of sun protection. Making young people more aware of the risks involved is of vital importance in preventing skin cancer. It is at this age when healthy practices should be consolidated. On average, the participants in our study are exposed to the sun for four hours a day, 10 months a year, or 1200 h of sunshine each year [21].

Reducing the duration of sun exposure and using more effective measures of photoprotection habits would help prevent diseases caused by UV radiation. For example, the use of sunscreen, and its subsequent reapplication when necessary, would be a very effective prevention strategy for participants in outdoor sports.

Previous studies have shown that participants in outdoor sports are at greater risk of skin cancer than the general population [10,22,23,24,25]. Moreover, the incidence of sunburn in water sports is further increased by the reflection of sunlight from the water and by continuous contact with rapidly moving water, which causes photoprotective lotions to wash off [26,27].

Of course, the incidence of solar radiation is also determined by the type of skin and how easily it burns [18]. In this context, there are usually no significant differences between men and women, although some studies have reported that women are more aware of the need for photoprotection, and are more likely to make use of protective measures, such as sunscreen and protective headwear [28].

Furthermore, the reapplication of sunscreen is almost as important as the first use. For maximum effectiveness during water sports, these lotions should be reapplied after 30 min [29,30]. In sports such as surfing or windsurfing, sunscreen should be re-applied as frequently as possible, due to the impossibility of reapplying it at the time of practice.

In our investigation, for each of the three disciplines considered, the elite athletes in the study population were exposed to UV radiation for considerable periods of time during the last sports season. These findings are comparable with those reported for other outdoor sports, such as those practised on the beach [16].

On the other hand, in certain sports, due to their inherent characteristics or according to the regulations, competitors cannot make use of certain elements of photoprotection. For example, surfers cannot wear sunglasses, and some athletes have commented that contact with the water can lead to sunscreen entering the eyes, causing irritation. However, in the winter season wetsuits are commonly worn, and these provide complete photoprotection for the body; protective lotions are then only needed for the face, hands and feet. Nevertheless, subsequent reapplication is still of vital importance (it is not uncommon for these athletes to train for continuous periods of three hours or more).

In our study, the surfers were least likely to reapply sunscreen, citing the discomfort it caused and claiming that it made the skin more slippery, which impaired their sports performance (this justification was also mentioned by other athletes).

According to our review of the literature in this field, the correct application of sunscreen (factor > 30) reduces the incidence of skin lesions, such as actinic neoplasia or solar keratosis [31,32].

A recent study of photoprotection habits in skaters reported that 74.4% had suffered at least one sunburn episode in the last year [33]. This finding suggests that the protection used by the athletes involved in this type of sport (closely related to those studied in our study) is insufficient. Another study, of U.S. university athletes, reported that 84% had suffered sunburn while playing outdoor sports [21]. In another geographic area, Australia, it has been reported that 62.9% of the participants in outdoor sports (such as hockey, football, tennis, and surfing) have suffered sunburn [34].

Regarding the duration of sun exposure, professional athletes are often exposed to the sun for long periods, as observed in one study of surfers [35] and in another of players of beach handball [16].

The photoprotection measures taken and the degree of sun exposure experienced vary among different sports. In part, this is governed by the competition rules and applicable regulations; for example, the International Federation for beach volleyball requires that short-sleeved shirts and shorts be worn, despite the risk to which this exposes the players, while in the Ironman triathlon event, the application of creams to certain areas of the body is prohibited [36,37,38].

Despite the above considerations, the elite athletes analysed in our study made similar use of sunscreen lotions as elite kite surfers [39], and greater use than university footballers [40].

In summary, to our knowledge the present study is the first to be conducted of elite athletes in water sports to determine the sun exposure experienced and the level of photoprotection employed. The importance of focusing on these athletes is highlighted by the fact that water sports are enjoyed on the beaches and seas of five continents. According to the International Surf Association, there are currently about 23 million surfers worldwide.

Although our study is based on professional surfers, windsurfers, and sailors who have recently taken part in world championship competitions, these events attract large audiences and inspire many to enter the sport. Therefore, the venues for these events would be suitable locations for awareness-raising campaigns about the dangers of prolonged exposure to the sun without adequate photoprotection. It is vital to educate young athletes in this respect, to protect their health both in the short and in the long term.

The following study limitations should be acknowledged. Firstly, the photoprotection measures taken and the sun exposure experiences of these athletes were not observed directly, but were reported by the participants in the questionnaires they completed. Nevertheless, this methodology is commonly employed in the study of health-related behaviour. Furthermore, the questionnaire used in this case was designed by a group of experts and based on previously validated examples.

Secondly, the study was conducted with elite, professional athletes who had been competing in world championship competitions. Among this group, there were fewer women than men, possibly for financial reasons, i.e., the existence of unequal monetary compensation/rewards in terms of sponsorship, grants, wages, and prizes.

Another limitation concerns the number of participants in the study population. The inherent nature of elites (athletes in water sports, in our case) greatly restricts the population size available for analysis.

Further study in the field of water sports is needed to determine whether amateur athletes present similar patterns of behaviour to the professionals, in other disciplines as well as for those studied here, in order to examine habits, practices, and knowledge about solar protection and to measure the damage produced to the skin by long periods spent outdoors in practice and competition.

Another interesting area for future research would be to study the anatomical locations of sunburn episodes, to identify which parts of the body need most protection, as done in studies such as the one carried out by Downs et al. in kite surfing [41]. We also believe it important to examine the educational background of athletes, to examine whether this might influence the type and frequency of photoprotection used. In addition, a study with similar characteristics should be conducted with amateur athletes, in which case the study population would be much larger, and so the data obtained and the conclusions drawn would be more conclusive [42]. Finally, a study with solar dosimeters, to quantify the radiation received by this type of athlete, would provide valuable information. 

## 5. Conclusions

Our study shows that elite athletes in water sports often make inadequate use of photoprotection measures and suffer frequent episodes of sunburn. This places them at high risk of skin lesions. The study results obtained highlight the need to inform practitioners of this type of sport about the importance of protecting themselves from sunburn, in order to reduce the danger of skin cancer and to prevent premature skin aging.

We believe that the use of the images of popular athletes could increase the effectiveness of future awareness campaigns about the danger of prolonged exposure to the sun, inspiring young practitioners of these sports to employ appropriate measures of photoprotection.

Finally, the provision of more areas of shade for athletes waiting to compete and for the spectators at this type of event would contribute to reducing the incidence of damage to the skin from sun exposure. Therefore, due to the characteristics of these athletes (in particular, surfers or windsurfers) and the environment in which they practice, we can give a series of specific recommendations to improve their photoprotection habits.

- Wear rash guards designed for sun protection;
- Wear neoprene wetsuits;
- Use helmets that also block UV radiation or neoprene hats with front and back visors;
- Use sunscreen lotions or photoprotection sticks resistant to water, and reapply them as soon as possible and at most two hours after the start of the activity;
- Use shade between competition events.

## Figures and Tables

**Table 1:** Distribution of protective habits, stratified by type of sport and gender.

		Surfing		Sailing		Windsurfing	
		Men	Women	Men	Women	Men	Women
Protection *n* (%)	Inadequate	24 (43.6)	11 (45.8)	39 (48.8)	23 (69.7)	16 (44.4)	6 (50)
	Adequate	31 (56.4)	13 (54.2)	41 (51.2)	10 (30.3)	20 (55.6)	6 (50)

**Table 2:** Sun exposure, stratified by gender and sport.

		Men (*n* = 171)			*p*	Women (*n* = 69)			*p*
		Surfing	Sailing	Windsurfing		Surfing	Sailing	Windsurfing	
		(*n* = 55)	(*n* = 80)	(*n* = 36)		(*n* = 24)	(*n* = 33)	(*n* = 12)	
Sun exposure (h)									
Weekdays		4.18 ± 1.55	4.55 ± 1.19	4.03 ± 1.36	0.105	4.42 ± 1.32	4.33 ± 1.38	3.50 ± 1.68	0.159
Weekend		4.53 ± 1.46	4.54 ± 1.38	4.06 ± 1.33	0.192	4.75 ± 1.54	4.70 ± 1.38	3.92 ± 1.24	0.209

Exposure habits expressed as mean ± SD; *p*-values for exposure assess significant differences among sports.

**Table 3:** Sunburn in the last sporting season, stratified by gender and sport.

		Men (*n* = 171)			*p*	Women (*n* = 69)			*p*
		Surfing	Sailing	Windsurfing		Surfing	Sailing	Windsurfing	
		(*n* = 55)	(*n* = 80)	(*n* = 36)		(*n* = 24)	(*n* = 33)	(*n* = 12)	
Sunburn last sporting season (%)									
0		22	31	14	<0.001	21	21	17	0.230
1–2		31	50	64		54	39	75	
≥3		47	19	22		25	39	8	

Note: *p*-values for sunburns assess significant differences among sports.

**Table 4:** Average age of participants, stratified by photoprotection adequacy.

	Photoprotection	
	Adequate	Inadequate
Average age (SD)	23.28 (6.03)	20.69 (5.4)
Significance	*p* = 0.000	

Note: *p*-value assesses significant differences.

## Data Availability

Dataset available on figshare.com: doi 10.6084/m9.figshare.13604429.

## Supplementary Materials

The following are available online at https://www.mdpi.com/1660-4601/18/2/800/s1, File S1: SUN PROTECTION INVESTIGATION QUESTIONAIRE.
