# Supplementary material for: Sun Exposure during Water Sports: Do Elite Athletes Adequately Protect Their Skin against Skin Cancer?
Source: Int J Environ Res Public Health. 2021 Jan 19;18(2):800. doi: 10.3390/ijerph18020800 (PMC7832317; doi:10.3390/ijerph18020800)
Supplement: Supplementary file 1 [file ijerph-18-00800-s001.pdf]

SUN PROTECTION INVESTIGATION

Final core items – ADULTS

For each question listed, please select the one answer that is the best response to the question.

Male ○ Female ○ Age [|] Years in windsurf competition :________

Section 1. Sun habits.

1.In the summer, on average, how many hours are you outside per day between 10 AM and 4 PM... on weekdays (Monday-Friday)?

30 minutes or less ....○
31 minutes to 1 hour ....○
2 hours ....○
3 hours ....○
4 hours ....○
5 hours ....○
6 hours ....○

2.In the summer, on average, how many hours are you outside per day between 10 AM and 4 PM... on weekend days (Saturday & Sunday)?

30 minutes or less ....○
31 minutes to 1 hour ....○
2 hours ....○
3 hours ....○
4 hours ....○
5 hours ....○
6 hours ....○

3.In the past 12 months, how many times did you have a red OR painful sunburn that lasted a day or more?

0 ○ 1 ○ 2 ○ 3 ○ 4 ○ 5 or more ○

For the following questions, think about what you do when you are outside during the summer on a warm sunny day.

	Never	Rarely	Sometimes	Often	Always
4.How often do you wear **SUNSCREEN**?	○	○	○	○	○
5.How often do you wear a **SHIRT WITH SLEEVES that cover shoulders**?	○	○	○	○	○
6.How often do you wear a **HAT**?	○	○	○	○	○
7.How often do you stay in the **SHADE or UNDER AN UMBRELLA**?	○	○	○	○	○
8.How often do you wear **SUNGLASSES**?	○	○	○	○	○
**9.How often do you spend time in the sun in order to get a tan?**	○	○	○	○	○

10.What is the color of your untanned skin?

Very fair .... ○
Fair .... ○
Olive .... ○
Light brown ....○
Dark brown .... ○
Very dark ....○

Section 2. Skin examination.

11.Have you EVER had your skin cheked for skin cancer from head to toe by a health professional?

No .... ○ (Skip to question 13)
Yes .... ○

12.If Yes, what is the month and year when you last had your skin checkes from head to toe?

Month [|] / Year [| | |]

13.In the last 12 months, have you or a partner examined your entire body, including your back, for skin cancer?

No .... ○
Yes .... ○

14.If Yes, how many times?

[|]

Section 3. Windsurf habits.

15.On average, how many hours a day do you practise windsurfing?

[|]

16. When you are practise windsurfing, do you usually use sunscreen on your face?

No .... ○
Yes .... ○

17. When you use suscreen, ¿sun protection factor (SPF) used is 30 or more? No ...... Yes......
18. Do you re-apply sunscreen? No .... Yes........
